# Malnutrition and its determinants among older adults people in Addis Ababa, Ethiopia

**DOI:** 10.1186/s12877-020-01917-w

**Published:** 2020-11-23

**Authors:** Tadele Abate, Berhanu Mengistu, Asmamaw Atnafu, Terefe Derso

**Affiliations:** 1 Department of Human Nutrition, Institute of Public Health, College of Medicine and Health Sciences, University of Gondar, Gondar, Ethiopia; 2 Department of Health Systems and Policy, Institute of Public Health, College of Medicine and Health Sciences, University of Gondar, Gondar, Ethiopia

**Keywords:** Malnutrition, Determinants, Older adults

## Abstract

**Background:**

In Ethiopia, malnutrition among older adults is under detected and, neglected area because the entire problem related with aging considered as fate of aging. Also, older adults are often omitted from public health research. Literatures are scarce in Ethiopia; there is limited information in the current study area among older adults using Min Nutritional Assessment (MNA). Therefore, this paper investigated the prevalence of malnutrition and its predictors among older adults people aged 65 years or above in Addis Ababa, Ethiopia.

**Methods:**

A community-based cross-sectional study was employed from January 1 to march 30, 2020 in Addis Ababa. Nutritional status of the older adults was measured by using Min nutritional assessment (MNA). Data from 662 older adults were collected through face to face interviews using a structured questionnaire. Bivariable and multivariable logistic regression analysis was done to identify factors associated with malnutrition.

**Result:**

The prevalence of malnutrition among older adults was found to be 26.6%[95% Confidence interval (CI):22.8, 30.1]. Depression [Adjusted Odds Ratio (AOR) = 7.57 95%CI: 5.01, 11.45], being poor [AOR = 1.95 95% CI: 1.166, 3.25], occupation; daily laborer and guard [AOR = 2.78 95% CI: 1.12, 7.17] and being old old [AOR = 2.62 95% CI: 1.62, 4.25] were significantly associated with the higher odds of malnutrition.

**Conclusion:**

This study illustrated that considerably high proportion of older adults were malnourished in Addis Ababa. Socio-economic characteristics and depression were significantly associated with malnutrition. Therefore, nutritional and social support activities are essential for older adults, particularly of those who are socio-economically disadvantaged and advanced age. In addition, regular nutritional screening and management as well as behavioral interventions should be strengthened as a pillar component of therapeutic interventions. Specific nutritional requirements, at later stages of life is changing, and with the population aged over 65 years increasing in low-income countries like Ethiopia, the research in this field is likely to increase further.

**Supplementary Information:**

The online version contains supplementary material available at 10.1186/s12877-020-01917-w.

## Background

Old age is defined as people aged 65 years or above [1]. In fact ageing is an irreversible biological phenomenon [2]. Particularly, older adults are highly vulnerable to malnutrition [3]. Globaly, about 13–78% of the older adults are suffering from malnutrition [4] . If it is not prevented or treated in time, it could increase morbidity and mortality rates, thus becoming a problem of great personal, family and socio-health significance [5].

So far, malnutrition in older adults remains under detected, under-treated, under-resourced and leads to further weight loss, increased infections and delay in recovery from illness as well as increased hospital admissions and length of stay [6]. Obviously, malnutrition is multi-factorial, it could be due to inadequate food intake, illness or both that causes increased nutrient loss, poor nutrient absorption or both [7]. Previous studies have shown that socio-cultural, health and socio-economic characteristics are main significant determinants of malnutrition [8, 9]. High odds of malnutrition has been documented among female, older age [10], unmarried [11], illiterate [12], low income [13], and living alone [12] older adults. Moreover, reduction of income and physical in capability in older adults increases vulnerability to food insecurity, which in turn predisposes to malnutrition [8]. Geographic and psychosocial concerns, can also affect dietary behaviors and nutritional status [9].

Population aging is an emerging challenge in Africa [14]. Despite the increasing number of older adults, there has been little effort to fulfill their health and nutritional needs, and therefore they seek a great attention in Ethiopia [15, 16]. Ethiopia has been implementing policy, program and strategies such as; Food and Nutrition program (FNP), Immunization and Health Sector Transformation Plan i.e. focusing to improve children, adolescents and maternal health and nutrition [17–19]. However, malnutrition among older adults is underdetected and neglected area because the entire problem related with aging considered as fate of aging. Also literatures are scarce in Ethiopia; there is a limited information in the current study area among older adults using Min nutritional assessment (MNA) tool recommended by the European Society for Clinical Nutrition and Metabolism (ESPEN) and fitted for Ethiopian older adults [20, 21]. Investigating the prevalence and determinants of malnutrition in a disadvantaged segment of population is of a crucial importance to develop strategies to address the problem and provide holistic support for the older adults. Therefore, this study was aimed to assess prevalence of malnutrition and its associated factor among older adults in Addis Ababa, Ethiopia.

## Methods

### Study setting and design

A community based cross sectional study was conducted from January 1 to March 30, 2020 among older adults aged 65 years or above in Addis Ababa, Ethiopia. Addis Ababa is the capital city of Ethiopia with an area of 530 km^**2**^. Addis Ababa city administration is divided into 10 administrative sub-cities. According the Ethiopian Central Statistical Agency (CSA) data, the estimated total population is of 3.4 million; of these 116,549(3.4%) were estimated to be older adults [22].

### Study population and sampling procedure

All older adults aged 65 years or above who lived in Addis Ababa for at least 6 months were included in the study. Sample size was computed using epi-info version 7.6 considering the following assumptions; percent of outcome in the unexposed group = 14.8 and Crude Odds Ratio (COR) = 2.3 as of malnutrition among older adults in northwest, Ethiopia [23]; 95% level of confidence, 1:1 ratio and 80% power. Considering a10% contingency for non-respondents and using design effect of 2, minimum sample size of 682 was obtained. Regarding sampling technique, the total lists of older adults were obtained from health extension workers, Addis Ababa health office. First, three (3) sub cities (Bole, Yeka and Lideta) were selected randomly from the 10 sub cities. The numbers of older adults in each randomly selected sub cities were proportionated to the sample size. Then, older adults were selected by systematic random sampling technique. For households with multiple older adults individuals, one older adult was selected randomly.

### Data collection procedures and variable measurement

An interviewer administered structured questionnaire was used to collect socio-demographic, financial support, alcohol consumption, cigarette smoking, and health and nutrition characteristics of older adults through house to house visit. Nutritional status of the older adults was measured by using Min nutritional assessment (MNA) tool [24] as recommended by the European Society for Clinical Nutrition and Metabolism (ESPEN) [20],and the MNA has been standardized for older adults in the other part Ethiopia [21]. The Mini nutritional assessment tool consists of 18-items grouped into four: general assessment (lifestyle, medication, stress, mobility, neuro-psychological problems and skin lesion), subjective assessment (perceived health and nutritional status), dietary assessment (number of meals, food and fluid intake, and mode of feeding) and anthropometric assessment (weight, height, arm and calf circumference and weight loss) [24]. The data was collected by 10 trained diploma nurses, and the supervisors were three BSc holder health officers. Prior to the data collection, 2 day training was given on data collection tools, ethics and approach in the interviewing techniques.

Weight of the study participants was measured using a beam balance to the nearest 0.1 Kg without shoes and all heavy clothing, including jackets, jerseys, and belts. Weighing scale was checked against a standard weight for its accuracy on daily basis. Calibration was performed before weighing each study participant by setting it to zero. Height of the study participants was taken using a seca vertical height scale standing upright in the middle of the board. Participants were asked to take off their shoes and stand in Frankfurt plane (stand erect, and look straight in horizontal plain). The occipital (back of head), shoulder blades, buttocks, and heels were touched measuring board and height was record to the nearest 0.01 cm.

To determine malnutrition, first the MNA score was ranged from 0 to 30 points. Using the MNA score, malnutrition was defined by MNA score less than 17 points. Furthermore, nutrition status was stratified: normal nutritional status (24–30 points), risk of malnutrition (17–23.5 points) and malnutrition (points less than 17) [24].

Geriatric Depression Scale item 15 (GDS-15) was used to assess depression among older adults suggested by the Royal College of Physicians, the British Geriatric Society and the Royal College of general practitioners. Thus, depression was defined using a cut-off point greater than or equal to five [25]. Another variable**,** wealth status was computed based on the possession of household assets (i.e., refrigerator, sofa, bicycle, television, radio, and mobile telephone) was used as a proxy for socio-economic status using principal component analysis. Finally, wealth index was ranked into three; poor, medium and rich. The last variable, old age was categorized into three; young old (65–74 years old), middle old (75–84 years old) and old old (> = 85 years old) [1]. To maintain the quality of data: regular supervision, spot-checking, and reviewing the completed questionnaire on daily basis was carried out by the investigators and supervisors.

### Data analysis

Data was cleaned and entered into the Epi-Data version 4.6 statistical software and exported to SPSS version 20 statistical package for analysis. Before analysis, missing values and outliers were checked and corrected by cross checking with original questionnaire. Frequencies and proportions were used to summarize variables. Besides, frequencies and proportions of each variable were presented using tables and figures. Association between malnutrition and each categorical variable was assessed using the binary logistic regression model. Variables such as age, head of the house hold, educational status, occupation, depression, marital status and wealth index which were significant at *P*-value < 0.2 in the bi-variable analysis were entered into the multivariable analysis. Accordingly, age, occupation, depression, wealth index, head of the household, marital status and educational status were adjusted in the binary logistic regression model. The significance of association was determined at a *P*-value of< 0.05 in the multivariable analysis, while the strength of association was measured by Adjusted Odds Ratio with 95% confidence interval.

## Results

### Socio demographic characteristics

A total of 662 older adults were included in this study (with a response rate of 97%). The majority of study participants were in the age range between 65 and 74 years (72.2%) and retired (73.6%). Half of older adults had no formal education (52%) and lived with their children (54.1%)(Table 1).

**Table 1:** Socio-demographic characteristics of older adults in Addis Ababa

Socio-demographic	Frequency (n)	Percent (%)
**Age**		
young old (65–74 years)	511	72.2
middle old (75–84 years)	118	17.8
old old (> = 85 years)	33	5
**Sex of the respondent**		
Female	422	63.7
Male	240	36.3
**Religion of the respondent**		
Orthodox	503	76
Muslim	99	15
Protestant	44	6.6
Catholic	14	2.4
**Family size**		
< =5	547	82.6
> =6	115	17.4
**Marital status**		
Single/divorce/widowed	370	55.9
Married	292	44.1
**Education status**		
No formal education	344	52
Primary education	234	35.8
Secondary to higher education	81	12.2
**Occupation of the responden**t		
Retired	487	73.6
daily laborer and guard	58	8.8
Merchant (self-employed)	117	17.7
**The main sours of financial support**		
Family support	317	47.9
Pension	212	32
Organization or NGO support	133	2.3
**Head of the house hold**		
Father	318	48
Mother	329	49.7
Children/relatives	15	2.3
**With whom respondent lives**		
With children	358	54.1
With partner	254	38.4
Alone	35	5.3
Others^a^	15	2.3
**Wealth index**		
Poor	227	34.3
Middle	216	32.6
Rich	219	33.1
**Alcohol consumption**		
Yes	62	9.4
No	600	90.6
**Smoking cigarette**		
Yes	3	0.5
No	659	99.5

^a^Relatives

### Depression

The prevalence of depression was found to be 27.64%.

### Prevalence of malnutrition among elderly people

The prevalence of malnutrition among older adults was 26.6% [95% CI, 22.8, 30.1]. More than half of study participants had eaten three full meals a day (59.52%) and had no decrease in food intake (56.34%) for the past 3 months. On the other hand, majority of older adults (82.93%) did not consume selected consumption marker of protein like dairy product (milk, cheese and yoghurt), legumes, egg and poultry every day (Table 2).

**Table 2:** Nutritional status of older adults in Addis Ababa

Variables	Frequency	Percent (%)
**Malnutrition**		
Yes	176	26.6
No	486	73.4
**Has food intake declined over the past 3 months**		
Sever decrease in food intake	88	10.3
Moderate decrease in food intake	221	33.4
No decrease in food intake	373	56.3
**Weight loss during the last 3 months**		
Weight loss greater than 3 kg	61	9.2
Dose not know	335	50.6
Weight loss between 1 kg and 3 kg	48	7.3
No Weight loss	218	32.9
**Mobility**		
Bed or chair bound	22	3.3
Able to get out of bed /chair but does not go out	93	14
Goes out	547	82.6
**Has suffered psychological or acute disease in the past 3 months**		
Yes	63	9.5
No	599	90.5
**Neuropsychological problem**		
Sever dementia or depression	23	3.5
Mild dementia	171	25.8
No psychological problem	468	70.7
**Body Bass Index (BMI)**		
BMI less than 19	61	9.2
BMI 19 to less than 21	222	33.5
BMI 21 to less than 23	276	41.7
BMI 23 or greater	103	15.6
**Lives independently (not in nursing home or hospital)**		
Yes	662	100
No	0	0
**Takes more than 3 prescription drugs per day**		
Yes	172	26
no	490	74
**Pressure sores or skin ulcers**		
Yes	19	2.9
No	643	97.1
**How many full meal dose the client eat daily**		
1 meal	11	1.7
2 meal	257	38.8
3 meal	394	59.5
**Selected consumption markers for protein intake**		
**At least one serving of dairy products (milk, cheese, yoghurt) per day**		
**Two or more serving of legumes or eggs per week**		
**Meat,fish or poultry every day**		
If 0 or 1 yes	549	82.9
If 2 yes	106	16
If 3 yes	7	1.1
**Consume two or more serving of fruit or vegetables pre day**		
No	607	91.7
Yes	55	8.3
**How much fluid (water, juice, coffee, tea, milk..) is consume per day**		
Less than 3 cups	303	45.8
3 to 5 cups	321	48.5
More than 5 cups	38	5.7
**Mode of feeding**		
Unable to eat without assistance	22	3.3
Self –fed with some difficulty	101	15.3
Self – fed without any problem	539	81.4
**Self –view of nutritional status**		
View self as being malnourished	89	13.4
Is uncertain of nutritional status	379	57.3
View self as having no nutritional problem	194	29.3
**In comparison with other people of the same age, how does the client consider his /her health status**		
Not as good	139	21
Dose not know	161	24.3
As good	289	43.7
Better	73	11
**Mid –arm circumference (MAC) in cc**		
MAC less than 21	62	9.4
MAC 21 to 22	208	31.4
MAC greater than 22	392	59.2
**Calf circumference (CC) in cm**		
CC less than 31	281	42.4
CC 31 or greater	381	57.6

### Factors associated with malnutrition among elderly people

The result of multivariable analysis revealed that depression, occupation, wealth index and age were independently and significantly associated with malnutrition. The likelihood of being malnourished was higher among older adults with old old aged [AOR = 2.94, 95% CI: 1.29, 6.67] and poor wealth index [AOR =1.95, 95% CI: 1.66, 3.25] compared to their counterparts. The study also found that the odds of malnutrition was 7.5 times higher among older adults who had depression [AOR = 7.57, 95% CI: 5.01, 11.45] compared to older adults who had no depression. Furthermore, occupation; daily laborer and guard [AOR =2.77, 95% CI: 1.09, 7.06] were significantly associated with higher odds of malnutrition among older adults (Table 3).

**Table 3:** Factors associated with malnutrition among older adults in Addis Ababa

Variable	Malnutrition		COR (95%CI)	AOR (95%CI)
**Age**	**Yes**	**No**		
young old	103	408	1	**1**
middle old	54	64	3.34(2.19,5.09)	2.62 (1.62, 4.25)*
old old	19	14	5.37(2.60,11.08)	2.94 (1.29, 6.67)*
**Occupation**				
Retired	148	339	4.20 (2.19, 8.06)	2.15(0.98, 4.38)
Daily laborer and guard	17	41	3.99 (1.72, 9.25)	2.77 (1.09, 7.06)*
Merchant (self-employed)	11	106	1	1
**Depression**				
Yes	110	73	9.42 (6.36, 13.97)	7.57(5.00, 11.45)*
No	66	413	1	1
**Wealth index**				
Poor	73	154	2.25 (1.44, 3.53)	1.94 (1.16, 3.25)*
Middle	65	151	2.05 (1.3, 3.231)	1.66 (0.90, 2.81)
Rich	38	181	1	1
**Head of the house hold**				
Father	79	239	1	1
Mother	89	240	1.12(.78, 1.59)	0.73(0.39, 1.38)
Children/relatives	8	7	3.45(1.21, 9.83)	1.58(0.40, 6.19)
Marital status				
Married	66	226	1	1
Divorce/widowed	110	260	1.44(1.07, 1.06)	1.36(0.87, 2.11)
Education status				
No formal education	106	238	1.80(1.00, 3.27)	1.63(0.79, 3.32)
Primary education	54	183	1.19(0.64, 2.24)	1.33(0.64, 2.78)
Secondary to higher	16	65	1	1

*indicates significant at *p*-Value less than 0.05in the multivariable analysis

## Discussion

Malnutrition is a significant public health problem especially, in resource-limited setting like Ethiopia where food insecurity prevail [3, 4, 25]. However, malnutrition in vulnerable groups such as older adults have usually been ignored [26, 27]. Our study is the one to focus on malnutrition of older adults and has a crucial importance to develop strategies to address malnutrition and provide holistic support for the older adults. This finding show that more than one-fourth (26.6%) of the older adults were malnourished. Moreover, depression, occupation, wealth index and age were independent predictors of malnutrition. The current finding was consistent with local studies from Debre Markos town, Northwest Ethiopia (22.7%) [28] and Hawassa city, Southern Ethiopia (28.3%) [21].

However, the current finding was higher than local finding reported from Aykele town, Amhara region, Ethiopia (17.6%). The discrepancy might be due to the difference of nutritional assessment methods, the former study assessed nutritional status based on Body Mass Index (BMI); whereas the current study assessed nutritional status based on Mini nutritional assessment which is a better way to identify nutritional disorder in older adults [24, 29]. Since BMI does not take into account the structure of the body or the percentage and distribution of adipose tissue, both of which change as a person ages [30] and also does not reflect changes that may occur due to sarcopenia (loss of skeletal muscle mass) or decrease in stature (osteoporosis, degenerative changes in the vertebrae and vertebral discs thinning) [31]. Thus, BMI is masking important weight changes and resulting in the failure to recognize malnutrition in time [24]. This implies that MNA is a better proxy than BMI to determine the nutritional status of older adults [29]. Similarly, a higher prevalence of malnutrition was reported in this study compared to previous studies reported from South Africa (5.5%) [32],Sire Lanka (13.6%) [33] and Brazil (18.8%) [34]. This might be due to the difference in the living standards and socioeconomic status like economic power of the older adults. The present study was done among older adults living in low income country compared to the earlier studies. This could explain lower food purchasing power of older adults, which in turn results in the consumption of undiversified food items, and limit their dietary intakes. Low dietary intake may increase the risk of malnutrition. Also, cultural difference including religious differences among older adults could be another possible reason.

The result the adjusted analysis demonstrated that the odds of malnutrition were higher among oldest old (aged 75 years old or above) older adults as compared to young old (aged 65–74 years old). This finding was confirmed by a local studies done in Debre Markose [28] and Gondar [23], Ethiopia which states that oldest old were more likely to be malnourished than young old people. Obviously, increased age puts older adults in a catabolic state i.e. body decomposition [35]. The risk of malnutrition is higher as their functional status is deteriorated possibly because of age increases and chronic co-morbidities such as respiratory disease, arteritis, and dementia. Impaired mobility and co-morbidities are known to impair appetite thereby food intake of the older adults [36].

This study also illustrated that depression was significantly associated with higher likelihood of malnutrition. Also, higher odds of malnutrition were reported among depressed older adults from studies done in South Africa, India and Netherland [2, 32, 37]. This might be due to depression negatively influences the appetite, food in-take and reduced energy in-take, and this can lead to weight loss and increase the risk of malnutrition [38].

The likelihood of malnutrition was increased by 1.94 folds among older adults in the poor wealth category. This finding is congruent with the study done in Northwest Ethiopia [23] and Northern Italy [13]. Thus, in poor households food consumption is reduced because of economic reasons, this may severely impact the physical and health status of the individual [39]. Particularly, older adults with poor socio-economic status might not afford nutrient dense and protein rich food, as a result cereal-based monotonous diet is common in this group of people which again diminishes their body ability and increases the risk of fat and fat-free loss [8, 13].

Finally, this paper showed increased odds of malnutrition among daily laborer and guard older adults compared to merchant (self employed) older adults. The adverse effect of unemployment on malnutrition mainly operates through its impact on the household economic deprivation. Similarly, most of the older adults in Ethiopia are not empowered compared to the non- older adults, which indirectly influence their economic power to purchase nutritious food.

Limitation: This study addressed an important public health concern, malnutrition. However, the cross-sectional nature of this study could not show the causal relationship between the response and explanatory variables. Hence, the study included samples from community; the findings may not be generalizable to those who did live in humanitarian organization like Mekedonia. Moreover, the study is not free from recall bias as the measurement of dietary issues was relied on memory, nevertheless efforts, such as training of data collectors and supervisors and appropriate probing techniques were used to minimize this bias. Finally, self-reported nature of the questionnaire also limits the external validity of the study.

## Conclusion

This study illustrated that considerably high proportion of older adults were malnourished in Addis Ababa. Socio-economic characteristics (wealth index and occupation) and depression were significantly associated with malnutrition. Therefore, nutritional and social support activities are essential for older adults, particularly of those who are socio-economically disadvantaged and advanced age. In addition, regular nutritional screening and management as well as behavioral interventions should be strengthened as a pillar component of therapeutic interventions. At last, specific nutritional requirements, at later stages of life is changing, and with the population aged over 65 years increasing in low-income countries like Ethiopia, the research in this field is likely to increase further.

## Supplementary Information

**Additional file 1.**

## Data Availability

Data will be made available upon request to the primary author/corresponding author.

## Abbreviations

AOR: Adjusted Odds Ratio
BMI: Body Mass Index
COR: Crude Odds Ratio
CSA: Central Statistical Agency
ESPEN: European Society for Clinical Nutrition and Metabolism
GDS: Geriatric Depression Scale
MNA: Mini Nutritional Assessment
WHO: World Health Organization
