# Supplementary material for: Malnutrition and its determinants among older adults people in Addis Ababa, Ethiopia
Source: BMC Geriatr. 2020 Nov 23;20:498. doi: 10.1186/s12877-020-01917-w (PMC7684921; doi:10.1186/s12877-020-01917-w)
Supplement: Supplementary file 1 — Additional file 1. [file 12877_2020_1917_MOESM1_ESM.docx]

# English version questionnaire

Section 1: socio-demographic related question

| Sr.No | Question | Response |
| --- | --- | --- |
| 101 | Age of the older adults? | ____ Years |
| 102 | sex | 1. Male 2. Female |
| 102 | Who is the Head of the house hold? | 1. Father 2. Mother 3. Other ………………….. |
| 103 | What is your marital status? | 1. Single 2. Married 3. Divorce 4. Widowed |
| 104 | Total family size (how many person live in these house)? | ………………In number |
| 105 | (wife)What is your education status? | 1. No formal education 2. Primary education 3. Secondary education 4. Certificate and diploma 5. Higher education |
| 106 | (Husband)what is your education status? | 1. No formal education 2. Primary education 3. Secondary education 4. Certificate and diploma 5. Higher education |
| 107 | ( wife)What is your occupation? | 1. House wife 2. Pension 3. Private organization employ 4. Privet work (self-employ ) |
| 108 | (husband )What is your occupation? | 1. Pension 2. Private organization employ 3. Private work (self-employ ) 4. Other |
| 109 | What is your main source of financial support? | 1. Pension 2. Family support 3. Organization (NGO) support 4. Other |
| 110 | Do you smoke cigarette? | 1. Yes 2. No |
| 111 | Do you consume alcohol? | 1. Yes 2. No |
| 112 | How many times per week | ----------------------- |

# Section 2: Wealth index questionnaire

| Section 2: wealth index for urban | | |
| --- | --- | --- |
| Number | Question | Response |
| 201 | Who is the owner of the house? | 1. Me 2. Rental 3. Family 4. Other |
| 202 | What is the main material of the roof in your house? | 1. Corrugated sheet 2. Grass 3. Plastic sheet 4. Corrugated iron 5. Other |
| 203 | What is the main material of the dwelling floor in your house? | 1. Soil/sand 2. Wood 3. Ceramic tiles 4. Cement 5. Stone 6. Other |
| 204 | What is the main material of the exterior wall in your house? | 1. Soil/sand 2. Bricks 3. Cement blocks 4. Stone 5. Wood 6. Other |
| 205 | How many rooms are available in this house? | ………………. |
| 206 | How many rooms in this house are used for sleeping? | …………….. |
| 207 | What is the main source of drinking water for member of your household? | 1. Public tap stand 2. Piped to neighbor 3. Piped to yard/plot 4. Piped to dwelling 5. Bottle water |
| 208 | Do you have a separate kitchen | 1. Yes 2. No |
| 209 | What type of toilet facility does this household use? | 1. Water flush latrine 2. Ventilated improved pit latrine 3. No latrine 4. Other …………. |
| 210 | Dose the household have electric power? | 1. Yes 2. No |
| 211 | What type of fuel dose your households mainly use for cooking? | 1. Electricity 2. Wood 3. Charcoal 4. Biogas 5. Natural 6. Other |
| 212 | Does your household have the following materials? | 1. Fixed phone 2. Refrigerator 3. Radio 4. Television 5. Electric mitad 6. Modern bed 7. Other |
| 213 | Does any member of the household have the following resource? | 1. Bicycle 2. Bajaj 3. Motor cycle 4. Car 5. Gari 6. Other |
| 214 | Does any member of the household have mobile phone? | 1. Smart phone 2. Not smart 3. Not |
| 215 | What is the main source of income for the household? | 1. Agriculture 2. Monthly salary 3. Trade 4. Family support 5. Daily laborer 6. Other |
| 216 | Does any member of this house hold have a bank or microfinance saving account? | 1. Yes 2. No |

# Section 3: Mini nutritional assessment tools

**1 .Has food intake declined over the past 3 months due to loss of appetite, digestive problems, chewing or swallowing difficulties?**

0 = severe decrease in food intake

1 = moderate decrease in food intake

2 = no decrease in food intake

**2 .Weight loss during the last 3 months**

0 = weight loss greater than 3kg (6.6lbs)

1 = does not know

2 = weight loss between 1 and 3kg (2.2 and 6.6 lbs)

3 = no weight loss

**3 .Mobility**

0 = bed or chair bound

1 = able to get out of bed / chair but does not go out

2 = goes out

**4. Has suffered psychological stress or acute disease in the past 3 months?**

0 = yes 2 = no

**5 .Neuropsychological problems**

0 = severe dementia or depression

1 = mild dementia

2 = no psychological problems

**6. Body Mass Index (BMI) = weight in kg / (height in m^2^)**

0 = BMI less than 19

1 = BMI 19 to less than 21

2 = BMI 21 to less than 23

3 = BMI 23 or greater

**7. Lives independently (not in nursing home or hospital)**

1 = yes 0 = no

**8 .Takes more than 3 prescription drugs per day**

0 = yes 1 = no

**9 .Pressure sores or skin ulcers**

0 = yes 1 = no

**10. How many full meals does the client eat daily?**

0 = 1 meal

1 = 2 meals

2 = 3 meals

**11. Selected consumption markers for protein intake**

• At least one serving of dairy products (Milk, cheese, yoghurt) per day

• Two or more servings of legumes or eggs per week

• Meat, fish or poultry every day

0.0 = if 0 or 1 yes

0.5 = if 2 yes

1.0 = if 3 yes

**12. Consumes two or more servings of fruit or vegetables per day?**

0 = no 1 = yes

**13. How much fluid (water, juice, coffee, tea, milk...) is consumed per day?**

0.0 = less than 3 cups

0.5 = 3 to 5 cups

1.0 = more than 5 cups

**14 .Mode of feeding**

0 = unable to eat without assistance

1 = self-fed with some difficulty

2 = self-fed without any problem

**15. Self-view of nutritional status**

0 = views self as being malnourished

1 = is uncertain of nutritional state

2 = views self as having no nutritional problem

**16. In comparison with other people of the same age, how does the patient consider his / her health status?**

0.0 = not as good

0.5 = does not know

1.0 = as good

2.0 = better

**17. Mid-arm circumference (MAC) in cm**

0.0 = MAC less than 21

0.5 = MAC 21 to 22

1.0 = MAC greater than 22

**18. Calf circumference (CC) in cm**

0 = CC less than 31

1 = CC 31 or greater

Section 4: Anthropometric measurements

| Elderly people height | --------------cm |
| --- | --- |
| Elderly people weight | --------------kg |
| Elderly people mid upper arm circumference (MUAC ) | ---------------cm |
| Elderly people calf circumference(CC) | ---------------cm |
